# Molecular characterization of *Staphylococcus aureus *isolates causing skin and soft tissue infections (SSTIs)

**DOI:** 10.1186/1471-2334-10-133

**Published:** 2010-05-26

**Authors:** Dan Yao, Fang-you Yu, Zhi-qiang Qin, Chun Chen, Su-su He, Zeng-qiang Chen, Xue-qing Zhang, Liang-xing Wang

**Affiliations:** 1 Department of Respiratory Medicine, the First Affiliated Hospital of Wenzhou Medical College, Wenzhou325000, China; 2 Department of Laboratory Medicine, the First Affiliated Hospital of Wenzhou Medical College, Wenzhou.325000, China; 3 Division of Infectious Diseases, Department of Medicine, Hollings Cancer Center, Medical University of South Carolina, South Carolina, USA

## Abstract

**Background:**

*Staphylococcus aureus*, particularly methicillin-resistant *S. aureus *(MRSA), is an important cause of pyogenic skin and soft tissue infections (SSTIs). The aim of present study is to investigate the molecular characteristic of *Staphylococcus aureus *isolates isolated from the pus samples from the patients with purulent skin and soft tissue infections in Wenzhou, China.

**Methods:**

Between December 2002 and June 2008, a total of 111 nonduplicate *S. aureus *isolates were collected from the pus samples of the patients with SSTIs in a teaching hospital in Wenzhou, China. All the tested isolates were confirmed as *S. aureus *using a Staph SPA agglutination kit, Gram's stain and a Vitek-60 microbiology analyzer. The homology among the tested isolates was determined by pulsed-field gel electrophoresis (PFGE). Multilocus sequence typing (MLST) was used to determine the sequence types (STs) of the selected isolates. The genotypes of SCC*mec *were determined by a multiplex PCR in the MRSA isolates. Panton-Valentine leukocidin (PVL) genes and *mec*A were also determined by another multiplex PCR.

**Results:**

Among the 111 *S. aureus *isolates, 48 and 63 isolates were community-acquired and hospital-acquired respectively. Sixty isolates were confirmed as MRSA harboring *mec*A detected by PCR. A total of 32 PFGE clonal types were obtained by PFGE, with 10 predominant patterns (types A to J). Twenty-five different STs including ST398 and three novel STs were found among 51 selected isolates. The main STs were ST239, ST1018, ST59, ST7 and ST88. Of 60 MRSA isolates, SCC*mec *II, III, IV and SCC*mec *V were found in three, 50, three and two isolates, respectively. The positive rates of PVL genes in overall isolates, HA-isolates, CA-isolates, MRSA isolates and MSSA isolates were 23.4% (26/111), 20.6% (13/63), 27.1% (13/48), 21.7% (13/60) and 25.5% (13/51), respectively. Eight (33.3%, 8/24) of 24 CA-MRSA isolates and 5 (13.9%, 5/36) of 36 HA-MRSA isolates were positive for PVL genes. ST239-MRSA-SCC*mec*III and ST1018-MRSA-SCC*mec*III clones were found to be main clones and spread between community and hospital.

**Conclusion:**

*S. aureus *isolates causing SSTIs showed considerable molecular heterogeneity and harbored high prevalence of PVL genes. Clonal spread was responsible for the dissemination of the isolates of *S. aureus *associated with SSTIs.

## Background

Infections caused by *Staphylococcus aureus*, especially methicillin-resistant *S. aureus *(MRSA), are emerging as a major public health problem. MRSA has been associated with skin and soft tissue infections (SSTIs), endovascular infections, pneumonia, septic arthritides, endocarditis, osteomyelitis and sepsis [1]. Since the first European isolate of MRSA was detected in 1960s, MRSA has become a leading cause of nosocomial infections worldwide [1,2]. In recent years, making even more complex the understanding of the epidemiology is the widespread dissemination of community-associated MRSA (CA-MRSA) afflicting individuals with no apparent risk factors for hospital acquisition and often possessing a potential virulence factor, Panton-Valentine leukocidin (PVL) [2]. The carriage of PVL genes, pulsed-field gel electrophoresis (PFGE), staphylococcal cassette chromosome *mec *(SCC*mec*) typing and multilocus sequence typing (MLST) were performed to monitor the evolutionary process of pandemic clones [3-6]. However, PVL genes have been detected infrequently in hospital-acquired MRSA isolates (HA-MRSA) in the Western Europe [5,7,8]. During the last decade, several major pandemic clones have been identified, including Brazilian clone, Hungarian clone, Portuguese clone, New York/Japan clone, Pediatric clone, Iberian clone and UK EMRSA-16 [9]. In China, the data from previous studies showed that the widespread predominant clone among HA-MRSA isolates was the Hungarian clone (ST239 SCC*mec *III) [10,11]. However, the epidemiology of Chinese *S. aureus *isolates isolated from the patients with SSTIs is poorly documented. The aim of this study was to investigate the epidemiology of *S. aureus *isolates associated with SSTIs by a variety of molecular methods and epidemiological data in a teaching hospital in Wenzhou, China.

## Methods

From December 2002 to June 2008, a total of 111 non-duplicate *S. aureus *isolates were collected from the pus samples of the patients with SSTIs including lesions requiring incision and drainage or with spontaneously draining purulent fluid, carbuncles, furuncles, boils, cellulitis with purulent drainage, chronic ulcer and deep wounds in a teaching hospital in Wenzhou, China. The *S. aureus *isolates isolated from the patients with SSTIs with clinical signs and symptoms of infection including increased white blood cell counts, fevers, redness, swelling, and exudate, were considered for inclusion. The isolates isolated from the patients without clinical signs and symptoms of infection were considered for colonization and exclusion. The clinical isolates were confirmed as *S. aureus *using a Staph SPA agglutination kit (bioMe'rieux, Marcy l'Etoile, France), Gram's stain and a Vitek-60 microbiology analyser (bioMe'rieu, Marcy l' Etoile, France). The isolates, which were isolated from the patients who had no risk factors for nosocomial acquisition and no hospitalizations or nursing home residence within a year before hospital admission within 48 hours after hospital admission, were considered to be community-associated. The isolates, which were isolated from the inpatients with no infections before hospital admission and more than 48 hours after hospital admission, were determined as HA-isolates. All tested isolates were digested with lysostaphin (1 mg/ml) (Sangon, China) at 37°C for 30 min. All the DNA was purified following the instruction of the Genomic DNA Extraction kit (Sangon, China). The DNA was stored at -20°C and prepared for PCR detection. Chromosomal DNA was prepared from all the isolates and cleaved with 40 U *Xba*I (Sangon, China). Electrophoresis was performed on 1% agarose gels in 0.5 M Tris/borate/EDTA buffer on a CHEF-Mapper XA PFGE system (Bio-Rad, Hercules, CA) for 22 hours at 14°C, with run conditions of 6 V/cm, a pulse angle of 120 degrees and pulse times from 5 to 20 seconds. A λDNA ladder (Amersham Biosciences, Piscataway, NJ) was used as molecular mass marker and bands were stained with ethidium bromide (0.5 μg/ml) and photographed under UV light. PFGE profiles were interpreted by the criteria described previously [12]. MLST was performed as described previously and ST numbers were assigned according to the *S. aureus *database http://saureus.mlst.net/[3]. The SCC*mec *typing of MRSA isolates was performed under the conditions as described previously [6]. The SCC*mec *V isolates were further determined by the methods described by Zhang et al and Milheirico et al. [6,13]. Reference strains were MRSA NCTC 10442 (SCC*mec *I), MRSA N315 (SCC*mec *II), MRSA 85/2082 (SCC*mec *III), MRSA JCSC 4744 (SCC*mec *IV) and MRSA HS663 (SCC*mec *V). The presences of the *mec *A and PVL genes were determined as described previously [5].

Ethical approval was not needed for the present study

## Results

One hundred and eleven *S. aureus *isolates were isolated from the patients aged from 4 days to 80 years old with purulent SSTIs. Among the 111 *S. aureus *isolates, 48 CA-isolates were associated generally with the diseases of purulent dermatitis, burns, pemphigus, maternal breast abscesses, while 63 HA-isolates were mainly associated with surgical intervention, burns or other localized infections with the risk factors as follows: the histories of prior hospitalization in an acute-care setting, hemodialysis, enter via-catheterization, arteriovenous shunts, tracheostomy or surgery in the year preceding MRSA isolation. And all the patients with purulent SSTIs caused by *S. aureus *recovered under the treatment of antibiotic and appropriate nursing care. Sixty isolates were confirmed as MRSA determined by PCR for detecting *mec*A. Of 60 MRSA isolates, 36 and 24 isolates were hospital-associated and community-associated.

The 111 isolates were clustered into 32 PFGE clonal types by PFGE, with 10 predominant patterns (designated types A to J) accounting for 72 isolates. The most prevalent PFGE clonal type was type A (13 isolates). The isolates belonging to PFGE type E, H, I and J were found only in HA-isolates, while the isolates belonging to PFGE type A, B, C, D, F, and G were detected not only in HA-isolates but also in CA-isolates.

MLST was performed on representative isolates based on PFGE type and all PVL-positive isolates. A total of 25 different sequence types (STs) were found among 51 selected isolates, mainly including ST239, ST1018, ST88, ST59 and ST7 (Table. 1). Three novel STs including ST1349, ST1357 and ST1358, were first found in the present study. Twenty-one STs including novel ST1349 and ST1358 were found in CA-isolates, while 17 STs were found in HA-isolates. The most dominant ST was ST239 which was found in 20 HA-isolates and four CA-isolates. Twenty ST239 HA-isolates persisting in our hospital from 2003 to 2008 were found among PFGE types B, E, J and M isolates and four ST239 CA-isolates were detected among PFGE type B isolates in 2007. ST1018 was found to be the second common ST. Seven ST1018 CA-isolates and six ST1018 HA-isolates were found between 2003 and 2008. The ST of six HA-isolates and four CA-isolates was ST88. Nine of 11 ST59 isolates were HA-isolates isolates. ST88 was the most common ST in the PVL-positive isolates (7/26).

**Table 1:** Molecular characterization of 111 isolates.

						SCC*mec *type					
						
ST	No. of isolates	PFGE	No. of MRSA	No. of PVL-positive strain	No. of CA-isolates	I	II	II	IV	V	NT
239	24	B, E, J, M	19	4	4			19			
1018	13	A	10	2	7		1	9			
59	11	D, H	2	0	2			2			
7	10	C	2	2	3			2			
88	10	G, I	6	6	4			3	3		
188	6	F	1	0	4			1			
5	4	K, X	2	2	3		2				
25	3	N	2	2	3			1		1	
243	3	O	2	0	2			1			1
594	3	L	3	0	2			2		1	
72	2	R	1	1	0			1			
366	2	S	2	0	2			2			
398	2	NT	1	2	1			1			
837	2	OA, OD	1	0	2			0			1
944	2	Q	1	0	1			1			
1301	2	P	0	2	0						
86	2	V, W	1	1	1			1			
1	2	U	0	0	2						
15	2	T	0	0	1						
20	1	Y	1	0	1			1			
370	1	Z	0	0	0						
1019	1	OB	1	0	1			1			
1349	1	OC	1	1	1			1			
1357	1	OE	1	1	0			1			
1358	1	OF	0	0	1						

PFGE types concluded 32 types: A to Z and OA, OB, OC, OD, OE and OF; NT: non-typeable

Of 60 MRSA isolates, three, 50, three and two isolates harbored SCC*mec *II, SCC*mec *III, SCC*mec *IV and SCC*mec *V, respectively. The remaining two isolates were non-typeable by the multiplex PCR method used.

Twenty-six isolates were positive for PVL genes. The positive rates of PVL genes in overall isolates, HA-isolates, CA-isolates, MRSA isolates and MSSA isolates were 23.4% (26/111), 20.6% (13/63), 27.1% (13/48), 21.7% (13/60) and 25.5% (13/51), respectively. The prevalence of PVL genes among CA-MRSA isolates (33.3%, 8/24) was higher than that among HA-MRSA isolates (13.9%, 5/36).

On the basis of MLST and SCC*mec *typing, ST239-MRSA-SCC*mec *III clone was found to be the most prevalent clone among HA-isolates (19/63), while ST1018-MRSA- SCC*mec *III clone was more likely to be obtained among CA-isolates (7/48). ST239-MRSA- SCC*mec *III and ST1018-MRSA- SCC*mec *III clones were obtained both in CA-isolates and HA-isolates. ST88-MRSA-SCC*mec *IV clone was found in three HA-isolates. The ST of the two isolates which could not be digested with *Sma*I was documented as ST398. One ST398- CA-MRSA-SCC*mec *III isolate was detected from a patient with the infection of gluteal muscles in 2002. The other ST398 isolate, which was detected in a patient aged 48 with the surgery of renal transplantation, was HA-MSSA. Novel ST1349, ST1357 and ST1358 were found in one PVL-positive CA-MRSA-SCC*mec *III isolate, one PVL-positive HA-MRSA-SCC*mec *III isolate and one CA-MSSA isolate, respectively.

Two MRSA isolates harbor SCC*mec *V were community-associated.

## Discussion

PFGE type A, B, C, D, F, G and K isolates had penetrated both in hospital and community in specified years, indicating that *S. aureus *isolates associated with SSTIs were spreading between community and hospital in Wenzhou, China. Whereas PFGE type E isolates were spread only in hospital. Previous studies demonstrated that the most prevalent clones in China were Brazilian/Hungarian (ST239-MRSA- SCC*mec *III) and New York/Japan (ST5- MRSA-SCC*mec *II) clones and had unique geographic distributions [10,11,14]. Similar to previous studies, ST239-MRSA-SCC*mec *III clone was also found to be the most prevalent clone among *S. aureus *isolates associated with SSTIs both in community and hospital. In contrast to previous study, only two ST5-MRSA- SCC*mec *II isolates were found in the present study. Although ST1018-MRSA-SCC*mec*III clone was not widely reported before, the clone was predominantly detected among CA *S. aureus *isolates, ST88 was the most prevalent ST among PVL-positive *S. aureus *isolates in our previous study [15]. ST88 clone was also an important clone among *S. aureus *isolates causing SSTIs. We had reported the molecular characteristic of 10 PVL-positive *S. aureus *isolates associated with SSTIs from January 2005 to January 2006 [15]. The data of the present study included the six PVL-positive isolates associated with SSTIs in our previous study [15]. Because other four PVL-positive MRSA isolates including one ST398 isolate died, the data of these four isolates were excluded from the present study. Historically it has been reported that HA-MRSA isolates mainly harbor SCC*mec *I, II and III, whereas SCC*mec *IV and V are found mainly in CA-MRSA isolates [16,17].

In contrast to previous studies, SCC*mec *IV was found in HA-MRSA isolates and SCC*mec *III was found both in HA- and CA-MRSA isolates, indicating that MRSA isolates harboring SCC*mec *IV and MRSA isolates harboring SCC*mec *III can spread between hospital and community.

PVL was supposed to be an important virulent factor in CA-MRSA isolates and a strong epidemiological association between PVL genes and successful CA-MRSA lineages, especially with the disease of SSTIs [18-20]. We also found the high prevalence of PVL genes among both HA- and CA-isolates and that the prevalence of PVL genes in HA-isolates was almost as high as that in CA-isolates. However, there was higher prevalence of PVL genes in CA-MRSA isolates than that in HA-MRSA isolates. MRSA clonal type ST398 is usually associated with the infections of animals, especially pigs [21,22]. However, human infections caused by MRSA ST398 isolates have been reported in many countries [23,24]. PVL-positive MRSA ST398 isolates associated with human infections has been reported [15,25]. Two PVL positive *S. aureus *ST398 isolates including one MRSA isolate and one MSSA isolate were also found in the present study. These data indicate that immediate monitoring is required for the spread of MRSA ST398 isolates.

## Conclusion

*S. aureus *isolates causing SSTIs showed considerable molecular heterogeneity and harbored high prevalence of PVL genes. Clonal spread was responsible for the dissemination of the isolates of *S. aureus *associated with SSTIs.

## Competing interests

The authors declare that they have no competing interests.

## Authors' contributions

DY, SSH and CC performed the laboratory measurements. FYY and LXW made substantial contributions to conception and design, and revised the manuscript critically for important intellectual content. ZQQ performed the analysis and interpretation of data. ZQC, XQZ and CC participated in design and coordination. DY and FYY drafted the manuscript. All authors read and approved the final manuscript.

## Pre-publication history

The pre-publication history for this paper can be accessed here:

http://www.biomedcentral.com/1471-2334/10/133/prepub
